# Glaucoma-related Changes in the Mechanical Properties and Collagen Micro-architecture of the Human Sclera

**DOI:** 10.1371/journal.pone.0131396

**Published:** 2015-07-10

**Authors:** Baptiste Coudrillier, Jacek K. Pijanka, Joan L. Jefferys, Adhiraj Goel, Harry A. Quigley, Craig Boote, Thao D. Nguyen

**Affiliations:** 1 Department of Biomedical Engineering, Georgia Institute of Technology, Atlanta, GA, United States of America; 2 Structural Biophysics Group, School of Optometry and Vision Sciences, Cardiff University, Cardiff, United Kingdom; 3 Glaucoma Center of Excellence, Wilmer Ophthalmological Institute, Johns Hopkins University School of Medicine, Baltimore, MD, United States of America; 4 Department of Mechanical Engineering, Johns Hopkins University, Baltimore, MD, United States of America; Casey Eye Institute, UNITED STATES

## Abstract

**Objective:**

The biomechanical behavior of the sclera determines the level of mechanical insult from intraocular pressure to the axons and tissues of the optic nerve head, as is of interest in glaucoma. In this study, we measure the collagen fiber structure and the strain response, and estimate the material properties of glaucomatous and normal human donor scleras.

**Methods:**

Twenty-two posterior scleras from normal and diagnosed glaucoma donors were obtained from an eyebank. Optic nerve cross-sections were graded to determine the presence of axon loss. The specimens were subjected to pressure-controlled inflation testing. Full-field displacement maps were measured by digital image correlation (DIC) and spatially differentiated to compute surface strains. Maps of the collagen fiber structure across the posterior sclera of each inflated specimen were obtained using synchrotron wide-angle X-ray scattering (WAXS). Finite element (FE) models of the posterior scleras, incorporating a specimen-specific representation of the collagen structure, were constructed from the DIC-measured geometry. An inverse finite element analysis was developed to estimate the stiffness of the collagen fiber and inter-fiber matrix.

**Results:**

The differences between glaucoma and non-glaucoma eyes were small in magnitude. Sectorial variations of degree of fiber alignment and peripapillary scleral strain significantly differed between normal and diagnosed glaucoma specimens. Meridional strains were on average larger in diagnosed glaucoma eyes compared with normal specimens. Non-glaucoma specimens had on average the lowest matrix and fiber stiffness, followed by undamaged glaucoma eyes, and damaged glaucoma eyes but the differences in stiffness were not significant.

**Conclusion:**

The observed biomechanical and microstructural changes could be the result of tissue remodeling occuring in glaucoma and are likely to alter the mechanical environment of the optic nerve head and contribute to axonal damage.

## Introduction

Glaucoma is an optic neuropathy characterized by the progressive loss of retinal ganglion cell (RGC) axons, accompanied by structural changes to the optic nerve head (ONH). The mechanisms leading to axonal degeneration in glaucoma are still unclear. Mechanical deformation of the scleral and ONH tissues is thought to play an important role in the disease [1–3]. Excessive pressure-induced deformation of the lamina cribrosa (LC), the connective tissue of the ONH, may trigger a series of biochemical events that eventually lead to axonal dysfunction and death. The mechanical environment of the ONH is determined by the structure and material properties of the different tissues of the ONH and the level of IOP [4, 5]. In addition, recent computational studies have demonstrated that scleral stiffness [5], geometry (radius and thickness) [6], and collagen fiber structure [7, 8] are important factors dictating the deformations of the LC. The objective of this study is to determine potential differences in mechanical behavior and collagen microstructure between scleras from donors with and without glaucoma.

There have been multiple attempts to evaluate how the mechanical response of the eye differed in patients with glaucoma. Most relied on measuring the structural stiffness of the entire globe, also known as “ocular rigidity”. Hommer et al. [9] measured a lower change in axial length caused by pressure change from pulsatil ocular blood flow in patients with open-angle glaucoma, which suggested an increased ocular rigidity. More recently, ocular rigidity was measured in patients with and without glaucoma using direct intraoperative cannulation [10]. No differences were observed between normal and glaucoma eyes. Although ocular rigidity measurements provide valuable information of the entire globe biomechanical properties, they cannot directly be used to determine scleral behavior. In contrast, *in vitro* testings of post-mortem scleral tissue allow the mechanical behavior of the sclera to be determined separately from that of the other ocular components. *In vitro* testings using animal models of glaucoma suggest that glaucoma is associated with an increased scleral stiffness. Using uniaxial tensile tests, Downs et al. [11] measured an increased equilibrium modulus in scleras of monkeys with experimental glaucoma. This finding was later confirmed by Girard et al. [12] using inflation testing, which requires less tissue preparation and allows the tissue to be tested under close to physiological conditions. Similar results were detected in mouse models of experimental glaucoma. In CD1 and B6 mice, exposure to chronic IOP elevation resulted in a stiffer deformation response [13]. We recently developed an inflation test to characterize the response of the human sclera to elevation of pressure [14, 15] and showed that the scleras of donors who had glaucoma deformed less in the peripapillary sclera [15], thus were “structurally stiffer”. Although structural stiffness depends on material properties of scleral tissue, scleral thickness and specimen geometry are confounded variables that may have contributed to the observed glaucoma related effects. The present study aims at relating pressure-induced scleral surface deformation measured in our inflation test to scleral material properties. Material properties are intrinsic to the scleral tissue and depend only on the microstructure of the sclera and the mechanical properties of its load-bearing constituents. Contrary to the structural stiffness, they do not depend on thickness or specimen geometry.

The microstructure of the sclera consists of a complex load-bearing network of collagen fibers embedded in a proteoglycan-rich matrix. The collagen fibers aggregate into lamellae that tend to be arranged within the plane of the sclera [16] and locally exhibit preferred orientations. In the recent years, researchers have investigated the potential effects of glaucoma on the collagen fiber structure of the human sclera using diffraction methods. Pijanka et al. [17] used wide-angle X-ray scattering (WAXS) to measure the through-thickness averaged preferred orientations and degree of alignment of collagen fibers of the posterior human sclera. Although no major differences in the dominant fiber directions were evident between glaucoma and non-glaucoma eyes, scleras from glaucoma donors exhibited a decreased degree of fiber alignment in the superior/temporal and inferior/nasal quadrants of the peripapillary sclera [17]. Using similar methods, spatial changes in collagen fibril anisotropy were also detected in the posterior sclera of mice with bead-induced chronic IOP elevation and axonal damage [18], indicative of dyamic remodelling of the scleral extracellular matrix in response to IOP elevation and/or glaucoma injury. Danford et al. [19] developed a method using small angle light scattering (SALS), which was applied to thin transverse sections and therefore could be used to detect depth variations in the collagen fiber structure. Significant differences in the preferred fiber directions and degree of fiber alignment (or eccentricity) were found when analyzing 1 cm^2^ pieces of sclera adjacent to the ONH. However, the effects of glaucoma were complex and varied among the different quadrants of the peripapillary sclera. The effects of experimental glaucoma on the fiber structure of the monkey sclera were indirectly measured by Girard et al. [12]. In this study, the authors inferred the anisotropy of the collagen structure from the measured displacement fields of an inflation test. The predicted fiber orientations and alignment showed no differences between the glaucoma and control eyes.

Glaucoma-related alterations to the mechanical properties and structure of the sclera likely change the biomechanical environment of the ONH. However, until recent advances in imaging technologies [20–22], the anatomical complexity and inaccessibility of the tissues of the ONH made the direct experimental measurements of these changes challenging. Instead, the deformation of the LC has been evaluated indirectly through computer modeling studies [4–8, 23]. An early approach, pioneered by Sigal and coworkers [5, 6, 24, 25], assumed a spherical geometry with uniform thickness and homogeneous isotropic linear elastic material properties for the sclera. Girard et al. [12, 23, 26] later created specimen-specific models of the monkey sclera and developed a mechanical model to describe scleral behavior, accounting for collagen alignment. A similar approach was adopted by Grytz et al. [27, 28] to determine the material properties of the human sclera from the inflation experiments of Fazio et al. [29]. However, in these studies, the collagen fiber structure was fitted to the experimental data rather than directly measured. We have developed an alternative modeling approach that uses specimen-specific collagen structure measured by WAXS [8]. Inverse finite element analysis is applied to determine the material properties of the collagen fibers and matrix constituents of the scleral tissues only. Here, we evaluate the collagen fiber structure and mechanical properties of the human sclera from donors with no history of glaucoma and donors diagnosed with glaucoma.

## Methods

### Overall approach

Using a standard inflation testing, we measured the displacement and strain response to elevation of intraocular pressure of the sclera from donors with and without glaucoma. We then subjected the specimens to wide-angle X-ray scattering (WAXS) to quantify the collagen fiber structure. We developed an inverse finite element analysis to estimate the material properties of the scleras by fitting a mechanical constitutive model to the experimentally measured displacements. The collagen micro-architecture, strain response, and material behavior were compared to evaluate the effects of glaucoma using mixed effects models accouting for spatial autocorrelation. The methods have been described in previous reports [8, 15, 17, 30] and are just briefly presented below.

### Ethic statement

We confirm that our research followed the tenets of the Declaration of Helsinki. As this study was performed using cadaverous tissue, it does not constitute human subjects research and is exempt from IRB review. The samples used in this study have been described in a previous publication [15].

### Specimens

Twenty-two human scleras from 7 donors with no history of glaucoma (9 eyes) and from 7 donors diagnosed with glaucoma (13 eyes) were obtained from the National Disease Research Interchange within 48 hours post-mortem (Table 1). In this study, eyes designated as diagnosed glaucoma were from donors whose medical records showed a coded diagnosis of glaucoma, who were prescribed known IOP lowering medications, and/or whose family confirmed that the deceased had been treated for glaucoma. To confirm the presence of glaucoma damage, 1 *μ*m thick optic nerve cross-sections of each specimen were assigned a quantitative grade between 0 and 3 [15, 31]. Diagnosed glaucoma specimens with axon loss greater than 25% were grouped into a category called “damaged glaucoma.” Diagnosed glaucoma specimens showing no optic nerve damage (axon loss lower than 10%) were called “undamaged glaucoma.” Some of the donors included in this study had diabetes.

**Table 1:** Donor information for the normal and diagnosed glaucoma scleras subjected to inflation testing and WAXS measurement of the collagen fiber structure. Grade 0 corresponded to an optic nerve with less than 10% of axon loss (normal appearance), grade 1 was 10% to 25% axon loss (mild damage), grade 2 was 25% to 50% axon loss (intermediate damage), and grade 3 was 50% to 75% axon loss (severe damage). In the specimen name, F stands for female, M for male, C for Caucasian, AA for African American, r for right, and l for left. Left/right eyes from the same donor are indicated with the same symbol in the specimen name.

Specimen	Age	Diabetes	Grade
**Non-glaucoma (normal) specimens**			
FC67r	67	yes	0
FC71r	71	no	0
FC74l	74	yes	0
FAA74l^‡^	74	yes	0
FAA74r^‡^	74	yes	0
FC77r^†^	77	no	0
FC77l^†^	77	no	0
MC77r	77	no	0
FC91r	91	no	0
**Undamaged glaucoma specimens**			
MC69r*	69	no	0
MC69l*	69	no	0
FC70r^§^	70	no	0
FC70l^§^	70	no	0
MAA71l^¶^	71	yes	0
MAA71r^¶^	71	yes	0
MC79l^††^	79	no	0
MC91l^‡‡^	91	no	0
**Damaged glaucoma specimens**			
MC79r^††^	79	no	2
FC81r**	81	no	3
FC81l**	81	no	3
MC82l	82	yes	3
MC91r^‡‡^	91	no	1

### Inflation testing

#### Displacement measurements

**Fig 1:**
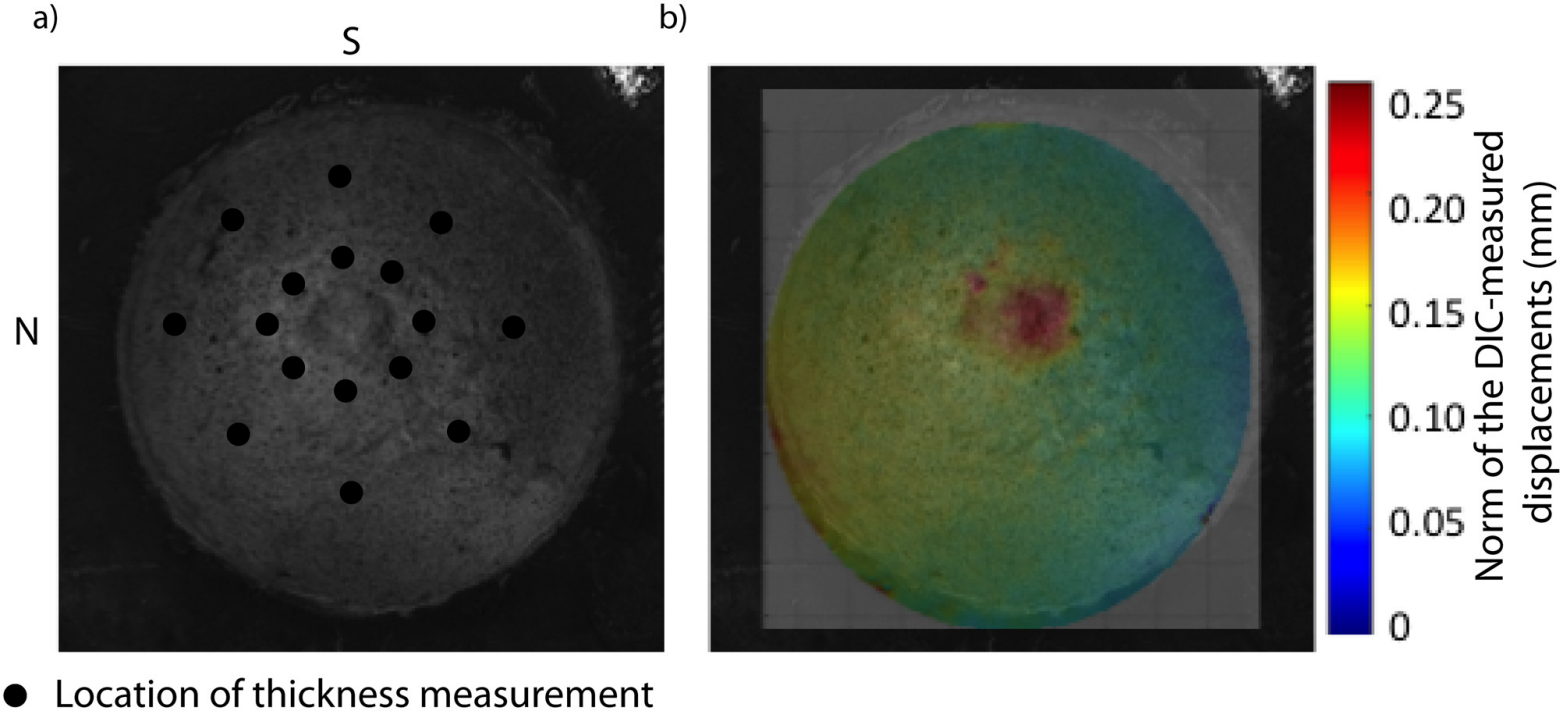
Inflation test. a) View of the specimen (FC81r) on the pressure chamber by one camera of the stereo system. Black dots indicates the location of the 16 thickness measurements. The ONH is centered. S stands for superior pole and N for nasal pole. b) Map of the norm of the DIC-displacements calculated at 30 mmHg superimposed over the camera view.

The inflation testing protocol was described in Coudrillier et al. [15]. Briefly, the specimens were cleaned of extra orbital fat and muscle, glued on a custom-made holder 3 mm posterior to the equator, and mounted on a pressure chamber enclosed by a humidity chamber. They were inflated through pressure-controlled injection of a saline solution. Pressure in the chamber was elevated from the baseline pressure of 1.5 mmHg to 30 mmHg at a rate of 1 mmHg/s. During inflation, two stereo cameras imaged the deforming sclera every 2 seconds. A stereoscopic DIC system (Vic3D, Correlated Solutions, Inc, Columbia, SC) with a 10 *μ*m uncertainty in the out-of-plane displacement [15, 32] was used to measured the 3-D displacement field of the scleral surface (Fig 1b). The DIC algorithm reconstructed the vertical positions (*Z*) of the undeformed surface and the three displacement components (*u*_*x*_, *u*_*y*_, *u*_*z*_) for a 2D cartesian grid parametrized by in-plane positions (X, Y) located every 140 *μ*m in both directions. Scleral thickness was measured at 8 locations in the peripapillary sclera and 8 locations in the midposterior sclera using an ultrasonic pachymeter (Fig 1a).

#### Finite strain calculation

**Fig 2:**
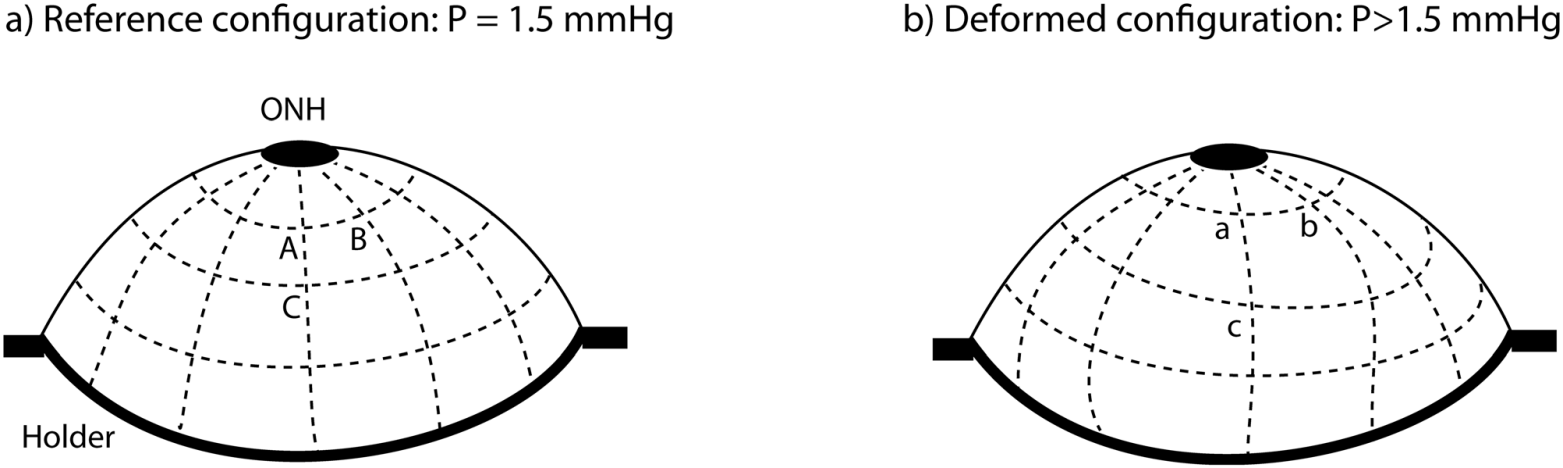
Finite strain calculation. a) Schematic of the scleral surface at the baseline pressure of 1.5 mmHg. The Z-positions of the surface were interpolated at the points of a polar grid, which was defined as a replacement of the DIC cartesian grid. The vector **AB** is along the circumferential direction, and the vector is **AC** is along the meridional direction. b) Schematic of the scleral surface in the pressurized configuration. The normal surface strains in the circumferential and meridional directions were calculated from the elongation of the vector **AB**, and **AC**, respectively.

Green-Lagrange strains were analytically calculated from the DIC displacements [8, 15]. Normal strains describe the elongation of a material line relative to the original length. In particular, we calculated normal strains in the circumferential direction (parallel to the scleral canal), and in the meridional direction (perpendicular to the scleral canal). The DIC-measured reference X and Y positions of the undeformed specimen were used to create a polar grid. The DIC-measured Z-positions and components of the displacement vector were interpolated to a polar grid centered on the ONH. This allowed for calculation of the deformed grid positions (**a**, **b**, **c**) corresponding to the undeformed positions (**A**, **B**, **C**) (Fig 2. The strains in the circumferential directions were calculated as the elongation of the vector connecting two points of the 3D grid corresponding to two circumferentially adjacent points on the polar grid: $E_{\theta \theta }=\frac{1}{2}[{\left(\frac{|\text{ab}|}{|\text{AB}|}\right)}^{2}-1]$, (Fig 2). Similarly, the strains in the meridional directions were calculated as the elongation of the vector connecting two points of the 3D grid corresponding to two radially adjacent points on the polar grid: $E_{\varphi \varphi }=\frac{1}{2}[{\left(\frac{|\text{ac}|}{|\text{AC}|}\right)}^{2}-1]$, (Fig 2).

### Wide-Angle X-ray scattering measurements of the fiber structure

**Fig 3:**
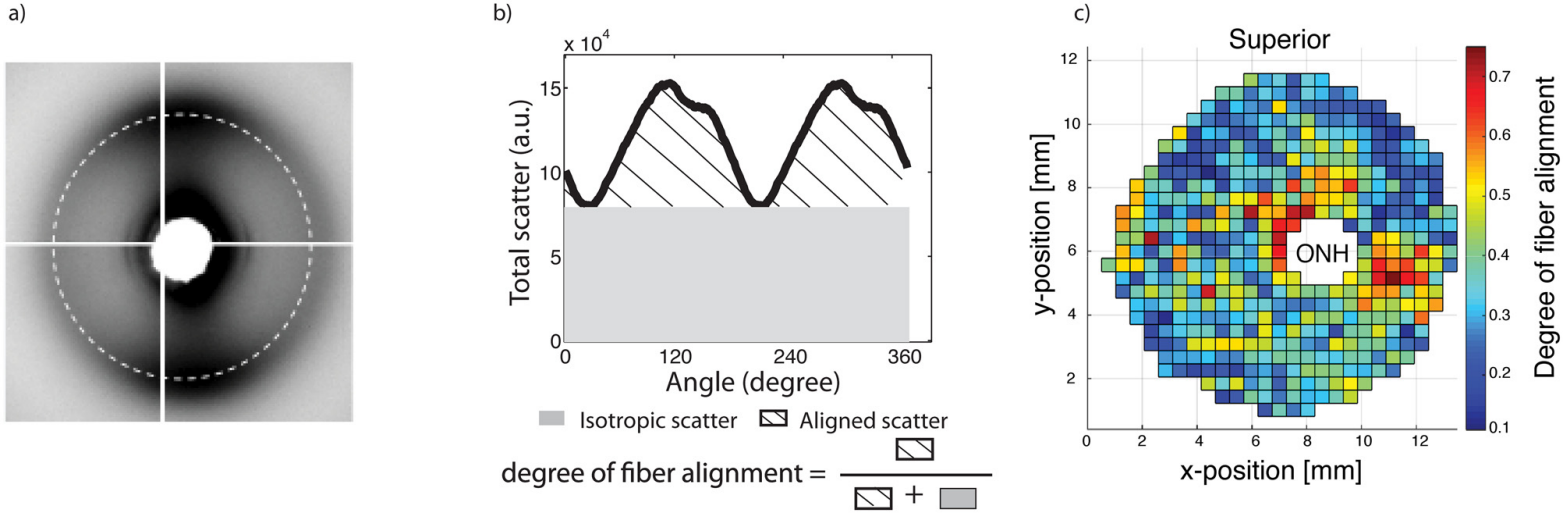
Collagen fiber structure measurement. a) Typical WAXS pattern. The circle (dashed line) corresponds to the collagen scatter. b) Angular intensity profile for a single WAXS measurements located in the peripapillary sclera. The degree of fiber alignment, which was defined as the ratio of the aligned scatter to the total scatter was calculated for each WAXS measurement and is mapped in c) for FC74l. (Reproduced with permission from Coudrillier et al. [30]).

Following mechanical testing, the specimens were immediately preserved in a 4% paraformaldehyde (PFA) solution until the time of X-ray measurements for characterization of the collagen structure. Wide-angle X-ray scattering (WAXS) patterns were recorded at 0.5 mm (horizontal) x 0.5 mm (vertical) intervals accross a 15 mm circular specimen, centered on the ONH, excised from each intact posterior sclera [17] (Fig 3). The WAXS pattern from scleral tissue was dominated by a well-resolved equatorial (i.e., perpendicular to the fiber axis) reflection from the regular 1.6 nm spacing of the constituent collagen molecules aligned near axially within the scleral fibers. Analysis of the WAXS patterns provides a quantitative measurements of bulk collagen fiber orientation, as an average value within the scleral volume sampled by the sclera. The collagen structure at one point of the sclera was described by the statistical distribution of collagen fibers *D*:

$$D\left(\Phi \right)=\frac{I(\Phi )}{\int_{0}^{\pi }I\left(\Phi \right)d\Phi },$$ (1)

where *I* is the total WAXS scatter, measured by increments of 1.4° in Φ. As defined, *D*(Φ)*d*Φ represents the number fraction of collagen fibers oriented between the angle Φ and Φ + *d*Φ. The degree of fiber alignment was calculated for every sampled point in the sclera by dividing the integral of the aligned scatter distribution by the corresponding integral of the total scatter, yielding a single value representing the proportion of fibers preferentially aligned at that point in the tissue. A spatial map of the degree of fiber alignment is shown in Fig 3c.

### Estimation of Mechanical Properties

#### Finite element model

We built a 3D specimen-specific finite element (FE) model of each posterior sclera based on the experimentally measured geometry and thickness [30]. Only the regions where WAXS patterns were collected were included in the FE model. We assumed that the sclera was in a stress-free state at the baseline pressure of 1.5 mmHg. Because the thickness profile of the sclera close to the ONH was unknown, the mesh was extended from the outer peripapillary sclera (2 mm from the center of the ONH) to the scleral canal with a generic model. This model assumed that the thickness of the peripapillary sclera linearly decreased from the experimentally measured thickness at the outer peripapillary sclera (Fig 1a) to the thickness at the scleral canal that was 0.4 mm for all specimens [33–35]. The DIC measured displacements were interpolated vertically to the surface of the generic peripapillary sclera model [30]. The ONH was not included in the model. Instead, we applied DIC-measured displacement kinematic boundary conditions at the scleral canal [30].

The sclera was modeled as a nearly incompressible, nonlinear elastic, and anisotropic material. The matrix, containing all non-fibrillar collagen constituents such as water, cells, elastin, and proteoglycans ensured that the sclera was incompressible. A neo Hookean model with shear stiffness *μ* was used to describe the matrix. The nonlinearity in the stress response of the sclera arose from the collagen fibers, which exhibit stretch-induced stiffening. This was mathematically described by an exponential function of the fiber stretch, which was parametrized by (*α*, *β*) such that 4α*β represents the axial stiffness of the fibers. The anisotropy of the sclera was caused by the local dispersion of collagen fibers, which was experimentally measured by WAXS. We assumed that all the collagen fibers were aligned in the plane of the sclera, which was defined using two vectors (**e**_*f*_, **e**_*p*_), where **e**_*f*_ represents the local preferred fiber orientation and **e**_*p*_ represents the direction normal to the local preferred fiber orientation in the scleral plane. Further, the dispersion of fiber in each element was described using the colocalized normalized WAXS scatter *D*(Φ) (Eq 1).

In summary, the strain energy density function for a given element of the mesh was:

$$W_{sclera}\left(C,X\right)=\frac{\mu }{2}\left(\bar{I_{1}}-3\right)+\frac{\kappa }{4}\left(I_{3}-\ln\left(I_{3}\right)-1\right)+\int_{0}^{\pi }\frac{\alpha }{\beta }\left[\exp\left(\beta \left(\lambda_{f}^{2}-1\right)\right)-\beta \lambda_{f}^{2}\right]D\left(\Phi ,X\right)d\Phi ,$$ (2)

where **C** is the Cauchy Green deformation tensor. The scleral material parameters (*μ*, *α*, *β*) were uniform across the scleral surface, while *D*(Φ) was position-dependent and informed by the co-localized WAXS experiments.

#### Inverse finite element analysis for parameter estimation

We recently presented an inverse finite element analysis (IFEA) to determine the material properties (*μ*, *α*, *β*) of each specimen given the DIC-displacement fields recorded at 15 pressure levels between 1.5 and 30 mmHg and the WAXS-measured collagen fiber structure [30]. A dense grid of parameter estimates (*μ*_*i*_, *α*_*j*_, *β*_*k*_) was generated, where (*i*, *j*, *k*) were simultaneously varied over a wide range of parameters spanning 2 orders of magnitude. For each parameter estimate, we simulated an inflation test replicating the experimental conditions, and computed the displacements at the nodes of the scleral surface. To determine the quality of the fit, a cost function was computed as the difference between experimental and finite element predicted displacement fields, integrated over the scleral surface and pressure loading. The solution of the IFEA, i.e. the material properties of the specimen, corresponded to the set of parameters (*μ*, *α*, *β*) minimizing the cost function over the tested parameter space.

### Statistical analyses

Since the data represent specimens nested within subjects and repeated measurements for each specimen, the effects of glaucoma diagnosis, peripapillary scleral quadrants, and history of diabetes on the degree of fiber alignment, peripapillary scleral strains, and material parameters were estimated using mixed linear models. The Bonferroni method was used to adjust pair-wise significance levels for multiple comparisons so that the experiment-wise error rate is ≤ 0.05. All analyses were performed using SAS 9.2 (SAS Institute, Cary, NC).

For each specimen, 17 measurements of degree of fiber alignment and 74 measurements of circumferential and meridional strains were available from each of the 4 peripapillary scleral quadrants. In order to look at the effect of peripapillary scleral quadrant on these measurements, a general linear mixed model was used, taking into account spatial autocorrelation among the measurements from each specimen as described in Fazio et al. [29].

## Results

### Collagen fiber structure in the peripapillary sclera

**Fig 4:**
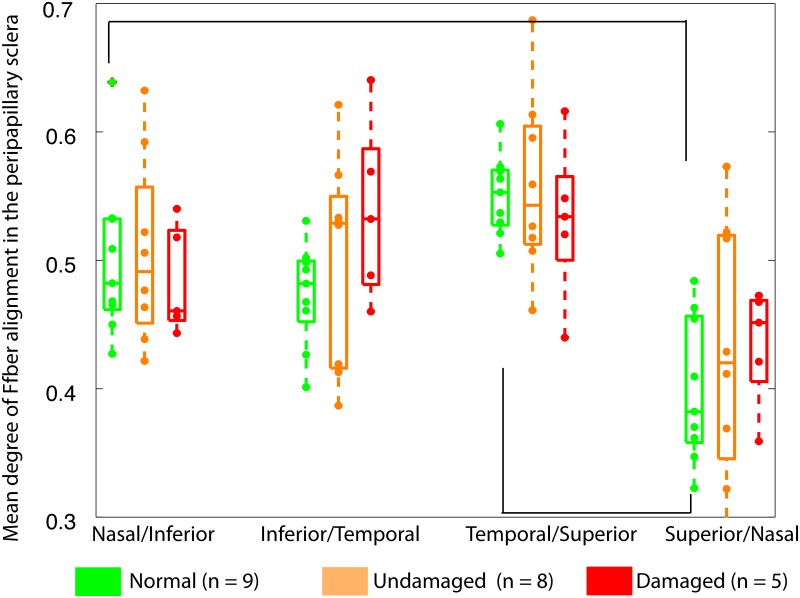
Collagen fiber alignment. Box plot of the average degree of fiber alignment in each of the 4 quadrants of the peripapillary sclera for normal, undamaged glaucoma, and damaged glaucoma specimens. The degree of fiber alignment was defined from the WAXS scatter intensity as the ratio of the aligned scatter to the total scatter (Fig 3). A scatter plot is superimposed to represent individual data points. Significant differences in degree of fiber alignment between two quadrants are indicated by connecting brackets (adjusted *p* ≤ 0.05).

All specimens presented strong circumferential alignment of collagen in the peripapillary sclera, independently of the glaucoma diagnosis. Table 2 presents the results of the statistical model for the degree of fiber alignment. The differences among the diagnosis groups were not statistically significant (*p* ≥ 0.05). For all specimens, the degree of fiber alignment was largest in the temporal/superior quadrant and lowest in the superior/nasal quadrant (Table 2 and Fig 4). Donors who had diabetes had a more anisotropic peripapillary sclera.

**Table 2:** Results of the general linear mixed model with spatial autocorrelation for the degree of fiber alignment in the peripapillary sclera with glaucoma diagnosis, quadrant, and diabetes history as explanatory variables. The variogram for the degree of fiber alignment increased and then leveled off as distance between 2 measurements of a same specimen increased, indicating spatial autocorrelation. S stands for superior, N for nasal, I for inferior, and T for temporal.

	Dependent Variable Estimated (Mean & 95% CI)	p-value	Category Different at Adjusted *p* ≤ 0.05
**Diagnosis**		0.69	none
Damaged	0.514 (0.474, 0.554)		
Undamaged	0.514 (0.481, 0.546)		
Normal	0.497 (0.468, 0.526)		
**Ppscl Quadrant**		<0.0001	TS,SN (<0.0001)
NI	0.518 (0.488, 0.547)		IT,SN (0.01))
IT	0.518 (0.489, 0.548)		NI,SN (0.002))
TS	0.537 (0.508, 0.567)		
SN	0.460 (0.430, 0.489)		
**Diabetes**		0.03	Yes, No (0.03)
Yes	0.531 (0.497, 0.566)		
No	0.485 (0.462, 0.508)		

We then included an interaction term between peripapillary sclera quadrant and glaucoma diagnosis. The interaction was borderline significant (*p* = 0.08), which motivated us to look at the effect of peripapillary scleral quadrant on fiber alignment within each diagnosis group. We found that the variations in degree of fiber alignment decreased as glaucoma diagnosis changes from normal to undamaged glaucoma to damaged glaucoma. Significant regional differences in degree of fiber alignment were only observed in the normal group when p-values were adjusted for multiple comparisons (Table 3). In comparison, the degree of fiber alignment in the peripapillary sclera was relatively uniform in both glaucoma diagnosis groups. In particular, there were no peripapillary scleral quadrants differing at *p* <0.05 in the damaged glaucoma group.

**Table 3:** Multivariate model to evaluate whether the effects of peripapillary sclera quadrant differed between glaucoma diagnosis for the degree of fiber alignment. Diabetes history was included in the model.

Diagnosis	Region	Estimated mean	p-value for the effects of quadrant by diagnosis	Regions differing at *p* ≤ 0.05 by diagnosis	Regions at adjusted *p* ≤ 0.05 by diagnosis*
**Damaged Glaucoma** (n = 5)	NI	0.49 (0.43, 0.55)	0.23	none	none
	IT	0.55 (0.49, 0.62)			
	TS	0.50 (0.44, 0.56)			
	SN	0.48 (0.42, 0.54)			
**Undamaged Glaucoma** (n = 8)	NI	0.53 (0.48, 0.57)	0.03	TS, SN (0.01)	none
	IT	0.50 (0.46, 0.55)		NI, SN (0.01)	
	TS	0.53 (0.49, 0.58)			
	SN	0.46 (0.41, 0.51)			
**Normal** (n = 9)	NI	0.50 (0.46, 0.55)	0.0001	TS, IT (0.05)	TS, SN (0.0001)
	IT	0.49 (0.45, 0.53)		TS, SN (<0.0001)	NI, SN (0.01)
	TS	0.54 (0.50, 0.58)		NI, SN (0.002)	
	SN	0.42 (0.38, 0.47)		IT, SN (0.02)	

*Bonferroni adjustment within glaucoma diagnosis. S stands for superior, N for nasal, I for inferior, and T for temporal.

### Inflation strain response

**Fig 5:**
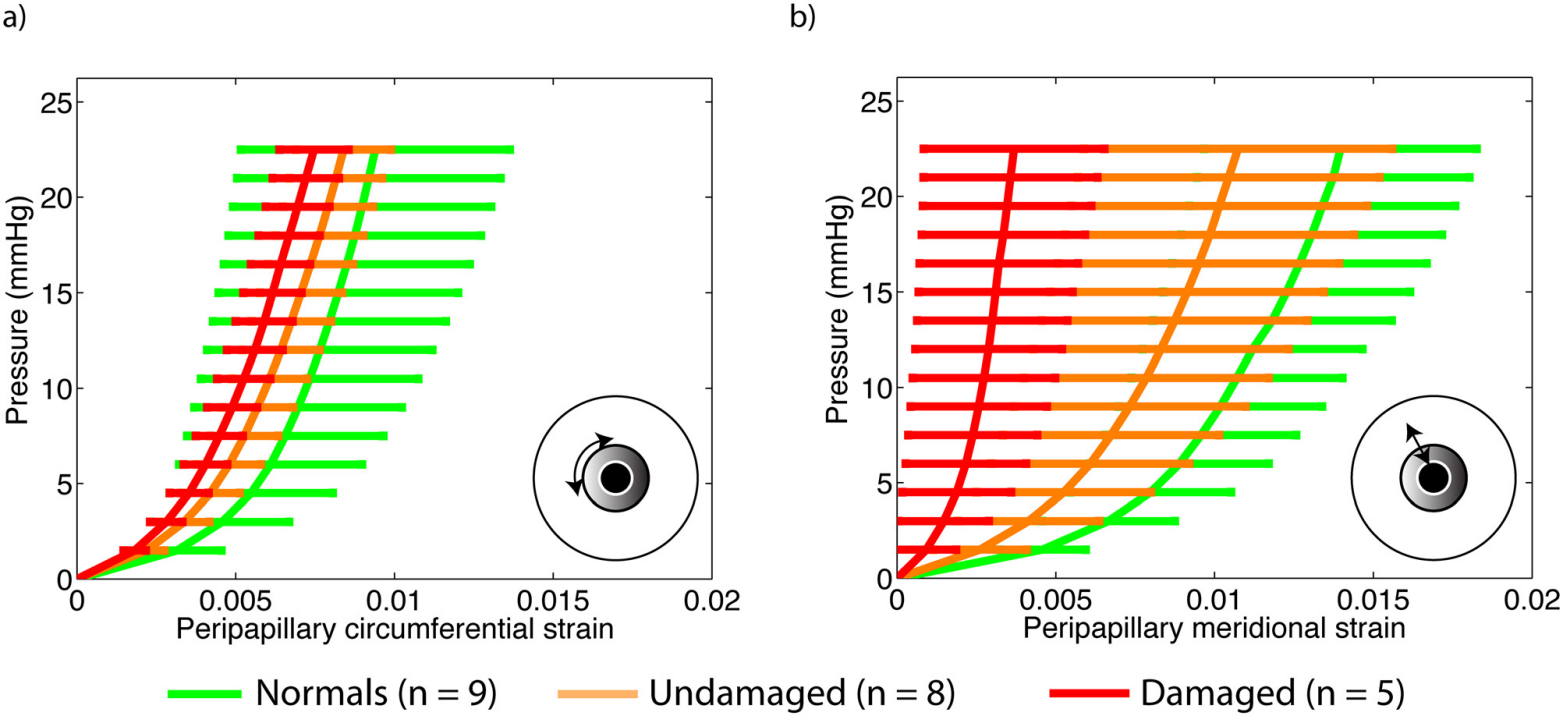
Inflation strain response. a) Pressure/average circumferential strain in the peripapillary sclera, b) pressure/average meridional strain in the peripapillary sclera.

Meridional strains in the peripapillary sclera were on average lower in eyes from donors diagnosed with glaucoma than in eyes from normal donors (Table 4). Damaged glaucoma had the stiffest inflation meridional and circumferential strain response. However, the differences in strains among the 3 groups were not significant (*p* = 0.95 and *p* = 0.30, Table 4). The circumferential and meridional strains averaged over the entire peripapillary sclera are plotted against inflation pressure in Fig 5. No differences in strain response were observed in the midposterior scleral among the 3 diagnosis groups.

**Table 4:** Results of the general linear mixed model for the peripapillary circumferential and meridional strains with glaucoma diagnosis, peripapillary scleral quadrant, and diabetes history as explanatory variables. The strains were calculated at 22.5 mmHg. For the two strain components, the variograms indicated that the measurements for a specimen were not spatially correlated. The 74 measurements from each specimen were assumed to have a compound symmetry correlation structure, in which any two measurements have the same correlation regardless of spatial location. S stands for superior, N for nasal, I for inferior, and T for temporal.

	CIRCUMFERENTIAL STRAIN PERIPAPILLARY SCLERA		MERIDIONAL STRAIN PERIPAPILLARY SCLERA	
	Mean & 95% CI	p-value	Mean & 95% CI	p-value
**Diagnosis**		0.95		0.30
Damaged	0.0091 (0.0030, 0.0153)		0.0060 (-0.0019, 0.0138)	
Undamaged	0.0103 (0.0051, 0.0154)		0.0079 (0.0007, 0.0151)	
Normal	0.0099 (0.0054, 0.0144)		0.0138 (0.0075, 0.0201)	
**Quadrant**		<0.0001		<0.0001
NI	0.0095 (0.0062, 0.0128)		0.0130 (0.0082, 0.0179)	
IT	0.0108 (0.0075, 0.0142)		0.0090 (0.0041, 0.0138)	
TS	0.0103 (0.0069, 0.0136)		0.0051 (0.0002, 0.0099)	
SN	0.0085 (0.0051, 0.0118)		0.0098 (0.0050, 0.0147)	
**Diabetes**		0.20		0.65
Yes	0.0119 (0.0064, 0.0173)		0.0103 (0.0048, 0.0157)	
No	0.0077 (0.0040, 0.0114)		0.0082 (0.0004, 0.0159)	

**Fig 6:**
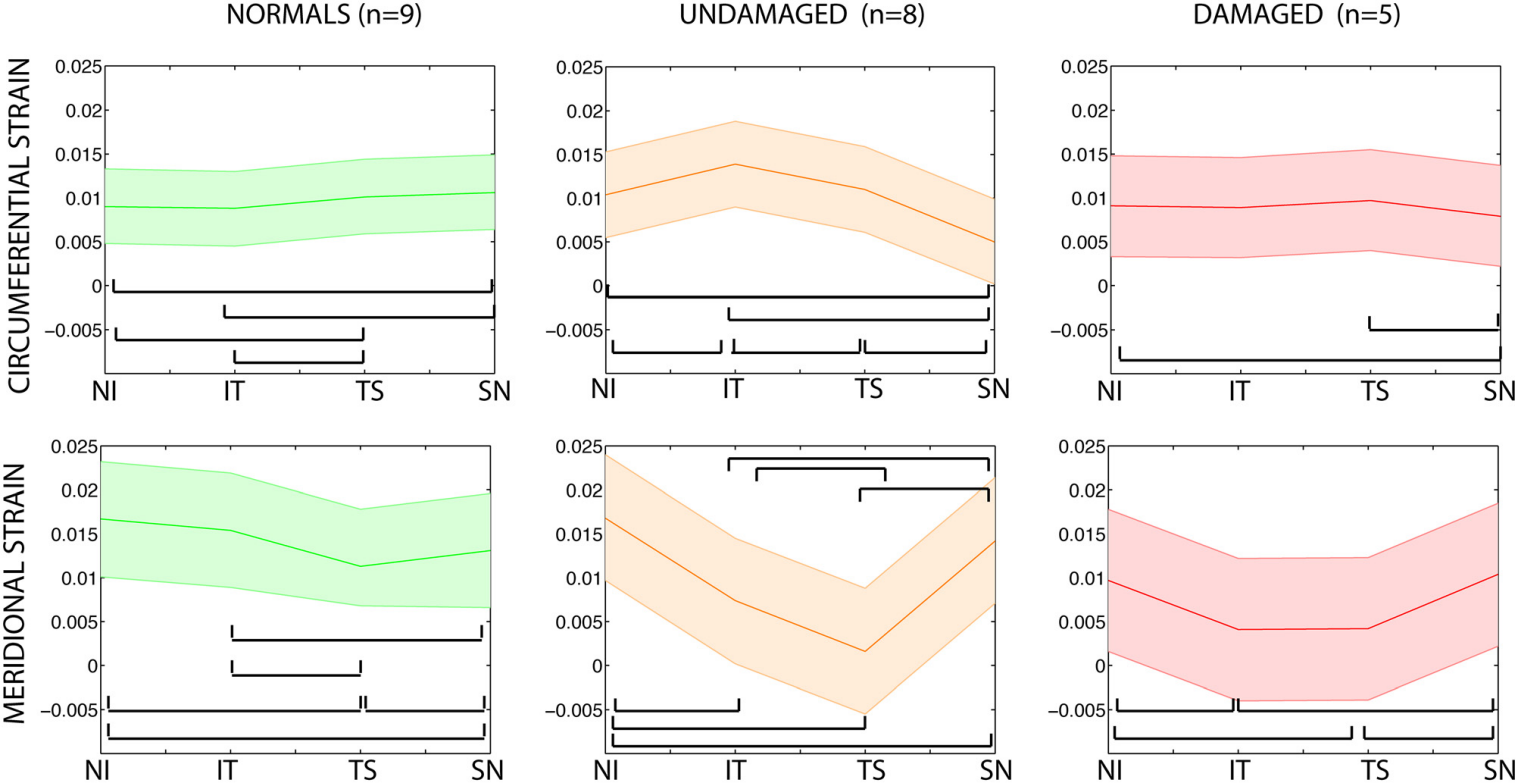
Strain profile in the peripapillary sclera. Regional variations in circumferential (top row) and meridional (bottom row) peripapillary scleral strains. Strains were calculated at 22 mmHg. Plotted is the mean (central line) and the 95% confidence intervals for the true mean (shaded regions). S stands for superior, N for nasal, I for inferior, and T for temporal. Significant differences in strain magnitude between two quadrants are indicated by connecting brackets (adjusted *p* ≤ 0.05).

We then added an interaction term between peripapillary sclera quadrant and glaucoma diagnosis to the previous model and found that the regional variations of circumferential and meridional strains in the peripapillary were significantly different among the three groups (*p* <0.0001). The regional patterns of peripapillary scleral strains are illustrated in Fig 6 for each group. The amplitudes of regional variations were largest among undamaged glaucoma specimens. Strain profiles in the peripapillary sclera were comparatively more uniform for normal specimens, although statistically significant for all 3 groups.

### Material parameters

The material parameters obtained after convergence of the inverse finite element analysis are reported in Table 5.

**Table 5:** Matrix modulus *μ*, parameters of the exponential fiber model (*α*, *β*) and fiber stiffness (4*αβ*) obtained by global optimization. On average, both matrix and fiber stiffness were larger for the damaged glaucoma group compared to the undamaged glaucoma group and larger for the undamaged glaucoma group compared to the normal group. Grade 0 represents ≤ 10% axon loss, 1 means 10–25% axon loss, 2 means 25–50%, and 3 means 50–75% axon loss.

		*μ*	*α*	*β*	Fiber stiffness: 4*αβ*
Grade	Specimen	kPa	kPa	-	MPa
**Non-glaucoma (normal) specimens**					
0	FC67r	175	10	100	4.00
0	FC71r	150	26.5	38	4.03
0	FC74l	300	15	141	8.46
0	FAA74l	470	40	42	6.72
0	FAA74r	670	1	105	0.42
0	FC77r	220	13.5	136	7.34
0	FC77l	225	15	145	8.7
0	MC77r	190	135	14	7.56
0	FC91r	250	2	137	1.10
**Average ± SD**		294 ± 170			5.37 ± 3.11
**Undamaged glaucoma specimens**					
0	MC69r	265	15	100	6.00
0	MC69l	330	27	74	7.99
0	FC70r	450	4	180	2.88
0	FC70l	725	3	240	2.88
0	MAA71l	300	55	40	8.8
0	MAA71r	190	222	16	14.2
0	MC79l	215	1.5	145	0.87
0	MC91l	1000	6	190	4.56
**Average ± SD**		435 ± 285			6.02 ± 4.26
**Damaged glaucoma specimens**					
2	MC79r	150	5	70	1.4
3	FC81r	225	21	113	9.49
3	FC81l	1200	175	17	11.9
3	MC82l	1350	17.5	180	12.6
1	MC91r	125	16	75	4.8
**Average ± SD**		610 ± 610			8.04 ± 4.80

On average, both matrix and fiber stiffness were larger for glaucoma specimens. The matrix stiffness was 294 ± 170 kPa for normals, 435 ± 285 kPa for undamaged glaucoma specimens, and 610 ± 610 kPa for damaged glaucoma specimens. The fiber stiffness was 5.37 ± 3.11 MPa for normals, 6.02 ± 4.26 MPa for undamaged glaucoma specimens, and 8.04 ± 4.80 MPa for damaged glaucoma specimens. However, the differences in matrix stiffness and fiber stiffness between the 3 groups were not significant as shown in Table 6. Although large variations in matrix and fiber stiffness were observed in the damaged glaucoma group, the eyes with more than 50% axon loss (FC81, MC82) had the largest fiber stiffness.

**Table 6:** Results of the general linear model for the glaucoma-related changes in matrix and fiber stiffness accounting for the effects of diabetes.

Outcome	Variable	Dependent Variable Estimated (Mean & 95% CI)	p-value
Matrix stiffness *μ* (kPa)	**Diagnosis**		0.37
	Damaged	618 (182, 1054)	
	Undamaged	438 (121, 756)	
	Normal	295 (13, 578))	
	**Diabetes**		0.93
	Yes	458 (74, 842)	
	No	443 (231, 654)	
Fiber stiffness *αβ* (kPa)	**Diagnosis**		0.43
	Damaged	8791 (3950, 13631)	
	Undamaged	7135 (2996, 11273)	
	Normal	5422 (1955, 8888)	
	**Diabetes**		0.40
	Yes	8171 (3216, 13126)	
	No	6060 (3256, 8864)	

**Fig 7:**
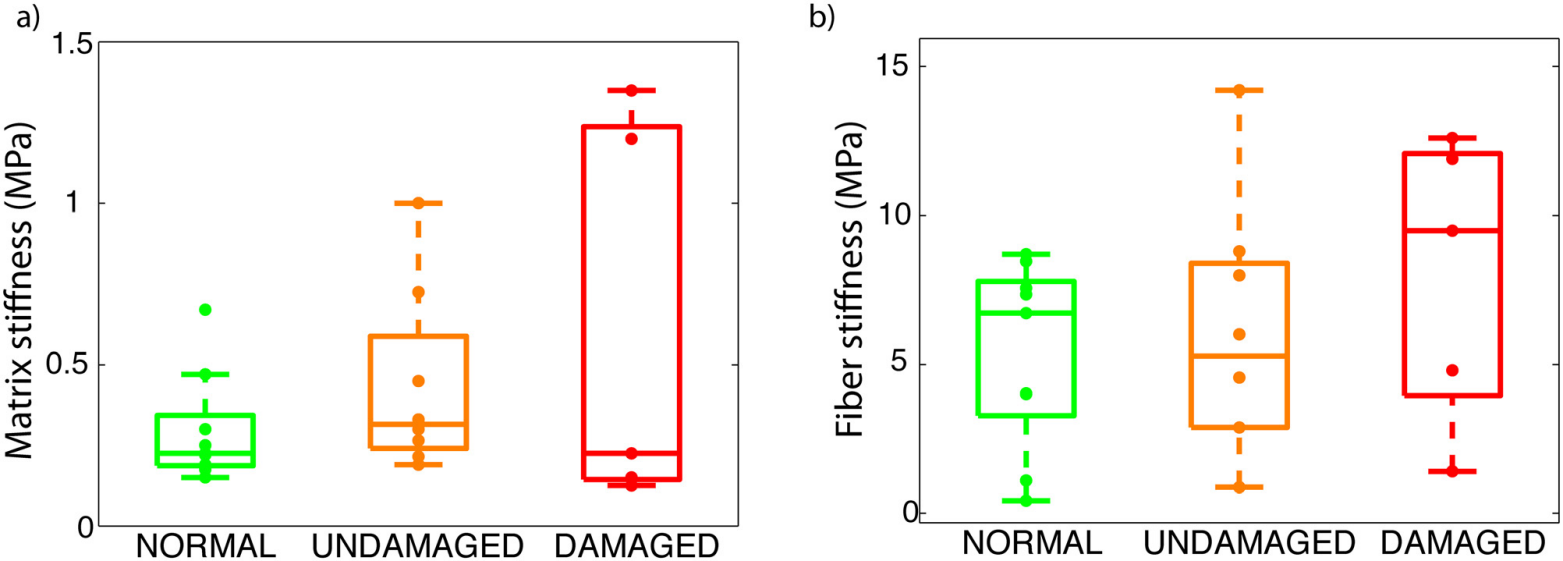
Material parameters. Box plot representing the matrix stiffness (a) and fiber stiffness (b) for normal, undamaged glaucoma, and damaged glaucoma specimens. Individual specimens are represented with a dot.

Fig 7 illustrates the effects of glaucoma diagnosis on the matrix stiffness *μ* and the fiber stiffness 4*αβ*

## Discussion

The objective of this study was to evaluate the potential effects of glaucoma on the collagen fiber structure and mechanical properties of the human sclera. We subjected 22 specimens to inflation testing and WAXS measurements of the fiber structure. Strains were directly calculated from the DIC-measured displacement fields of the inflation experiments and material properties were estimated by fitting a constitutive model to the experimental displacements. The observed differences between normal and diagnosed glaucoma sclera in structure and biomechanics were small in magnitude. The main findings of this study were:

- The peripapillary sclera of all specimens was characterized by a ring of circumferentially oriented collagen fibers. Regional patterns of degree of fiber alignment in the peripapillary sclera were different among the 3 groups. Glaucoma specimens showed less regional variations in the degree of fiber alignment around the ONH than normals. Normal specimens had a well-defined pattern of degree of fiber alignment, with the temporal/superior quadrant being the most anisotropic and the superior/nasal being the least anisotropic.
- On average, meridional (perpendicular to the scleral canal) strains were lower in damaged glaucoma specimens than in undamaged and normal specimens, but the differences were not statistically significant. The regional variations of tangential and meridional strains in the peripapillary sclera were significantly different among normal, undamaged glaucoma, and damaged glaucoma specimens. Undamaged glaucoma specimens showed the largest variations in strains in the peripapillary sclera. In contrast, normal specimens had a comparatively more uniform strain profile.
- Although the differences in material properties among the different groups were not statistically significant, matrix stiffness and fiber stiffness were on average lowest in the normal group, followed by the undamaged glaucoma group and the damaged glaucoma group. The three eyes with severe axonal damage had a fiber stiffness twice of the average fiber stiffness seen in normal specimens.

We recently investigated the effects of glaucoma on the collagen fiber structure of the human sclera [17], and reported that the degree of collagen fiber alignment (or fiber anisotropy) was significantly lower in the superior-temporal and inferior-nasal peripapillary scleral quadrants and noticeably but not significantly larger in the inferior temporal quadrant in glaucoma eyes compared with non-glaucoma eyes. The overall pattern of less regional variation in the peripapillary sclera for glaucoma specimens is in agreement with the current study. Decreased collagen alignment was later reported in a mouse model of experimental glaucoma [18]. The present study included 8 of the 12 specimens from Pijanka et al. [17] and 14 additional specimens. Although the relative sectorial differences in fiber alignment among diagnosis groups were not significant, the regional variations in degree of fiber alignment around the ONH showed significant differences consistent with previous observations, which may have important biomechanical implications. Computer modeling [7, 8, 26] showed that variations in the degree of fiber alignment in the peripapillary sclera determine the level of mechanical strains within the ONH. Therefore, the observed differences may contribute to changes in the biomechanical environment of the ONH, which may play a role in axonal dysfunction and degeneration. To date, there is no consensus on the effects of glaucoma on the collagen fiber structure of the human sclera. A recent study using SALS confirmed that glaucoma affected the collagen fiber structure throughout the scleral thickness, but the effects were complex and slightly different than those reported here [19]. The fiber splay, an alternative definition of the degree of fiber alignment, differed between normal and glaucoma specimens only in the temporal quadrant, where normal specimens had a more aligned fiber structure than glaucoma specimens. Further studies applying WAXS to scleral sections could be used to characterize depth variations in the collagen structure, and should help better estimate the effects of glaucoma [36].

In a recent inflation test of the human sclera [15], we compared the peripapillary and midposterior scleral pressure/strain response of 12 normal, 6 undamaged, and 7 damaged scleras of age 75 and older. We found that the meridional strain response in the peripapillary sclera was significantly lower for glaucoma specimens compared with non-glaucoma specimens. In the present study, we measured a similar glaucoma-related stiffening of the meridional direction in the peripapillary sclera. This finding was not statistically significant here because we only used a subset of the specimens from our previous inflation study [15] that were also subjected to WAXS measurements and also included other normal and undamaged glaucoma specimens of age younger than 75. Structural stiffening of the sclera with glaucoma was also measured in humans *in vivo* and mice *in vitro*. Hommer et al. [9] observed an increased ocular rigidity in patients with open angle glaucoma. This can most likely be attributed to an increased structural stiffness of the sclera as this tissue is the main load bearing structure of the eye. Nguyen et al. [13] measured a stiffer inflation response following exposure to elevated IOP in a mouse model of experimental glaucoma [13]. In our study, strains and collagen alignment were not simply correlated. Normal specimens, which showed strong regional variations in the degree of fiber alignment in the peripapillary sclera had relatively uniform strain profiles in this region. In contrast, glaucoma specimens, which had a relatively uniform collagen structure in the peripapillary sclera, showed strong variations in their strain profiles. Other important factors controlling the magnitude of strain such as geometry and thickness of the peripapillary sclera may explain why the sectorial variations of strains do not closely match those of the degree of fiber alignment.

We conducted the present modeling study to better understand the individual contribution of scleral material properties, collagen structure, and geometry to the observed differences in the inflation response of glaucoma specimens. Our model included a specimen-specific representation of the scleral collagen structure, thickness, and geometry. By fitting our constitutive model to the experimental displacement fields, we were able to separately estimate the effects of glaucoma on the stiffness of individual ECM constituents. Our findings suggest that both the matrix and fiber stiffness are larger in glaucoma. Glaucoma eyes had on average a greater matrix and fiber stiffness than normal eyes. Although the increases in fiber and matrix stiffness were not statistically significant, the stiffening closely followed the extent of glaucomatous damage, with the normal eyes being the most compliant followed by the undamged glaucoma eyes, and the damaged glaucoma eyes. Interestingly the scleras of the two donors with the largest axonal damage (FC81 and MC82) had the stiffest collagen fibers. The 2 other damaged glaucoma specimens (MC79r and MC91r) were not abnormally stiff but the contralateral eye of those donors did not show any axon loss. We can speculate that MC82 and FC81 may have had the disease for a longer period of time, and that any remodeling effects associated with glaucoma would be larger in those eyes. We would need to obtain medical records documenting glaucoma diagnosis and treatment to confirm this hypothesis. Previous studies using a non-human primate model of experimental glaucoma have also showed that exposure to elevated IOP resulted in increased material properties. Downs et al. [11] found that scleras of monkey eyes exposed to elevated IOP had a larger equilibrium modulus than the contralateral control scleras as measured in a uniaxial tensile test. Girard et al. [12] later subjected normal and experimental glaucoma monkey eyes to inflation testing and reported an increased scleral tangent modulus in the experimental glaucoma eyes.

We are currently investigating the microstructural changes occuring in glaucoma that may cause an increase in fiber and matrix stiffness. Fiber stiffening could occur through the accumulation of nonenzymatic glycation type cross-links of collagen fibrils. This process occurs naturally in the aging sclera [37] but could be accelerated by glaucoma. In our constitutive model, the matrix contained all the non-collagen components, i.e. elastin, proteoglycans, cells, water. Extensive remodeling of the extra-cellular matrix has been reported in the LC of glaucoma eyes [38–41]. Several studies have shown that the remodeling also extends to the sclera. Electron microscopy studies have indicated a reduction in collagen density in the human glaucoma peripapillary sclera [42]. Knepper et al. [43] showed that the chondroitin sulfates content was larger in the anterior sclera (close to the schlemm canal) in open-angle glaucoma eyes. Our laboratory recently conducted experiments where we depleted the sclera from its glycosaminoglycans (GAGs) [44] and found that changes in proteoglycans and GAG composition of the sclera have a strong effects on the inflation response. It remains unknown whether proteoglycan and GAG composition are altered in glaucoma in the posterior sclera.

Below, we discuss the limitations of our methodology. The weaknesses associated with the use of DIC for displacement and strain calculation have been addressed in previous publications [8, 15, 30, 32]. Displacements were computed with an inherent uncertainty of 8 *μ*m [15]. We estimated the strain resolution to be 0.07% [32], which was in agreement with that reported in a recent inflation study of mouse arteries using 3D DIC [45]. Strains values in the peripapillary sclera were typically larger than 0.5% and we are confident that the glaucoma-related stiffening effects seen in the peripapillary sclera are not due to excessive noise in the strain measurements. However, it would be beneficial to replicate this work with other methods such as speckle-based interferometry [12, 29], ultrasonic measurements [46, 47], or sequential DIC [48] that have been shown to have a better displacement resolution than DIC.

Second, we assumed that the matrix and fiber stiffness were uniform across the sclera. In other words, a collagen fiber of the peripapillary sclera had the exact same diameter, crimp angle than one in the midposterior sclera and the cross-link density was uniform. Quigley et al. [42] showed that the collagen fibers in the peripapillary sclera had on average a lower diameter than those in the midposterior sclera. In addition, collagen crimp period was recently shown to significantly vary over the corneoscleral shell of the sheep eye (Jan et al., IOVS 2014, ARVO E-abstract 3715-A0229). Our assumption also implied that the matrix was identical between the peripapillary and midposterior sclera. However, proteoglycan [49] and elastin [39] composition varies with scleral locations. For instance, the peripapillary sclera contains elastin oriented circumferentially around the ONH [39]. Yet, elastin is almost not present in the midposterior sclera [42]. The variations in matrix composition would most likely contribute to spatial variations in the matrix stiffness that were ignored in our model. In a recent study characterizing the shear behavior of the bovine sclera, Argento et al. [50] showed that the in-plane shear modulus was 50% larger in the peripheral sclera than in the peripapillary sclera. Assuming position-dependent matrix and collagen fibers stiffness in our model would have considerably increased the computational cost of the inverse method may have led to the existence of multiple minima to the cost function. Our model also assumed that collagen fibers were oriented in the plane of the sclera. Using multiphoton microscopy, Pijanka et al. [17] showed that the collagen fiber structure in the peripapillary sclera was not entirely planar. In the inner one-third of the peripapillary sclera, interwoven lamellae were observed crossing at random angles out of the scleral plane.

Another important limitation was the relatively low sample size (22 specimens) of this study, which may have affected the statistical significance of our findings. WAXS studies of hydrated (and thus weakly scattering) biological tissues are only possible using high-intensity X-rays provided by national synchrotron facilities, to which access is limited due to high cost and demand, thus limiting the number of specimens we could analyze. The study was also limited by the lack of clinical information on the donors. We confirmed the degree of glaucomatous damage of all donor eyes that were used in this study by qualitative assessment of axonal loss. Many of the diagnosed glaucoma donors showed no optic nerve damage. Because elevated intraocular pressure is an important risk factor for the disease, patients with ocular hypertension are often misdiagnosed as glaucoma. This study also included eyes from diabetic donors. Diabetes was found to have significant effects of matrix stiffness, and age-related stiffening of the sclera [51]. We included diabetes diagnosis in the statistical model. The effects of diabetes were significant only for the degree of fiber alignment, which was larger for diabetic donors. Because of the few number of eyes from donors who had diabetes and glaucoma, we did not include an interaction term between glaucoma and diabetes in the models and cannot draw definitive conclusions on the combined effects of diabetes and glaucoma on scleral mechanics.

WAXS only yields a through-thickness averaged measurement of the collagen fiber structure. However, the collagen alignment is not uniform throughout the scleral thickness. Danford et al. [19] used SALS to quantify collagen fibril orientation across 70 *μ*m-thick serial sections of scleral tissue and showed that the collagen alignment was strongest in outer sclera. This finding was recently confirmed by WAXS by Pijanka et al. [36]. Based on the recent computational modeling findings of Petsche et al. [52], we expect the through-thickness variations in collagen structure to have minimal effects on the inflation response of the midposterior sclera [52]. The WAXS measurements were collected at 0.5 mm intervals, and we assumed that the collagen structure measured at the closest point to the scleral canal extended to the LC. A recent study showed that highest collagen alignment was located at a 0.5 mm distance from the LC [53, 54]. It is possible that our model did not capture this local variations in the collagen structure along the scleral canal, which were showed to have strong effects on ONH biomechanics [8, 54]. However, it is unlikely that these local variations in collagen structure would influence the results of the IFEA for the scleral properties.

Another difficulty is inherent to post-mortem *in vitro* testing. We are unable to determine whether the observed changes associated with glaucoma are evidence of a response to the disease or preceded the development of the disease. Studies addressing this research question have produced conflicting outcomes. On the one hand, increased stiffness was observed in both monkey [12] and mouse [13] models of experimental glaucoma suggesting that the observed changes are a response to the disease. Further, mice with an induced mutation in collagen 8A2 exhibited a stiffer scleral inflation response at baseline and less axon loss with chronic IOP elevation [55]. On the other hand, experimental scleral cross-linking was found to increase glaucoma damage in a mouse model [56], suggesting that having a stiffer sclera is a risk factor for developing glaucoma. In addition, Morris et al. [47] measured larger IOP spikes in eyes with stiffer scleras, providing more evidence that increased scleral mechanical stiffness may be detrimental in glaucoma. Measuring the biomechanical properties of the living eye would help distinguish between those two alternative explanations. This has become a topic of great clinical interest as altering ocular biomechanics was suggested as a possible therapy for glaucoma [57, 58]. Sigal et al. [25] recently developed an indirect method to predict scleral stiffness from OCT-measurable parameters, which could be used in a longitudinal study of scleral mechanics.

In conclusion, we compared the mechanical properties and the collagen fiber structure of 13 scleras from donors diagnosed with glaucoma and 9 scleras from non-glaucoma donors. The regional variations in fiber anisotropy in the peripapillary sclera were different between glaucoma and normal specimens, with glaucoma specimens displaying a more homogenous structure. On average, glaucoma specimens were stiffer: they had a larger matrix, a larger fiber stiffness, and lower meridional strains in the peripapillary sclera. Although these differences were not statistically significant, the extent of the differences increased with glaucomatous damage. The differences in mechanical properties of the sclera may increase the susceptibility of the ONH to glaucoma damage and should be investigated further.
